# Highly Efficient Capture of Volatile Iodine by Conjugated Microporous Polymers Constructed Using Planar 3- and 4-Connected Organic Monomers

**DOI:** 10.3390/molecules29102242

**Published:** 2024-05-10

**Authors:** Chaohui Li, Qianqian Yan, Huanjun Xu, Siyu Luo, Hui Hu, Shenglin Wang, Xiaofang Su, Songtao Xiao, Yanan Gao

**Affiliations:** 1 Key Laboratory of Ministry of Education for Advanced Materials in Tropical Island Resources, Hainan University, No 58, Renmin Avenue, Haikou 570228, China; lich@hainanu.edu.cn (C.L.); yanqianqian@hainanu.edu.cn (Q.Y.); 2021220856000114@hainanu.edu.cn (S.L.); sxf@hainanu.edu.cn (X.S.); ygao@hainanu.edu.cn (Y.G.); 2 School of Science, Qiongtai Normal University, Haikou 571127, China; xuhuanjun86@iccas.ac.cn; 3 China Institute of Atomic Energy, Beijing 102413, China; xiao200112@163.com

**Keywords:** conjugated microporous polymers, topology, iodine adsorption, porous materials, nuclear energy

## Abstract

The effective capture and recovery of radioiodine species associated with nuclear fuel reprocessing is of significant importance in nuclear power plants. Porous materials have been proven to be one of the most effective adsorbents for the capture of radioiodine. In this work, we design and synthesize a series of conjugated microporous polymers (CMPs), namely, TPDA–TFPB CMP, TPDA–TATBA CMP, and TPDA–TECHO CMP, which are constructed based on a planar rectangular 4-connected organic monomer and three triangular 3-connected organic monomers, respectively. The resultant CMPs are characterized using various characterization techniques and used as effective adsorbents for iodine capture. Our experiments indicated that the CMPs exhibit excellent iodine adsorption capacities as high as 6.48, 6.25, and 6.37 g g^−1^ at 348 K and ambient pressure. The adsorption mechanism was further investigated and the strong chemical adsorption between the iodine and the imine/tertiary ammonia of the CMPs, 3D network structure with accessible hierarchical pores, uniform micromorphology, wide π-conjugated structure, and high-density Lewis-base sites synergistically contribute to their excellent iodine adsorption performance. Moreover, the CMPs demonstrated good recyclability. This work provides guidance for the construction of novel iodine adsorbent materials with high efficiency in the nuclear power field.

## 1. Introduction

Over the past few decades, nuclear power has come to be considered one of the most important sources of green and clean energy in the world [1,2]. However, the safe disposal and proper management of radioactive waste generated from the spent nuclear fuel remain a great challenge. Among various nuclear wastes, radionuclide ^129^I (15.7-million-year half-life) and ^131^I have been identified as the two most significant volatile waste species, as their quick diffusion into air and environment would cause radiological contamination in terms of both the global environment and biological health [3,4,5]. In fact, an increased incidence of thyroid cancer was observed following the Chernobyl and Mayak accidents [6]. Therefore, the development of novel materials to effectively dispose of these nuclear wastes has become an extremely important and urgent task.

By far, adsorption is regarded as an effective strategy for radioiodine removal due to advantages such as easy operation, high efficiency, low cost, and so on. In recent years, various adsorbent materials have been developed for iodine capture, including inorganic zeolites [7,8], activated carbon [9,10], and silver-doped mordenite [11], as well as organic–inorganic hybrid materials (mainly referring to metal–organic frameworks, MOFs [12,13,14,15]), and organic porous framework materials such as covalent organic frameworks (COFs) [16,17,18,19,20], conjugated microporous polymers (CMPs) [21,22,23,24], porous aromatic frameworks (PAFs) [25,26,27], porous organic frameworks (POFs) [28], hyper crosslinked polymers (HCPs) [29,30], etc. Among them, organic porous framework materials appear to be one of the most effective adsorbents for iodine capture [5].

As a special class of organic porous frameworks, CMPs, combine extended π-conjugated structures with permanent micropores and have attracted much attention due to their unique electronic properties, structural modularity, high porosity, and good thermal and chemical stability [31]. Organic monomers with three or more cross-linking sites and rigid structures are needed to construct CMPs. The monomers are connected through covalent bonds and the networks extend along a three-dimensional (3D) direction, leaving voids between the rigid monomers, and thus giving CMPs porosity [32]. As a result, CMPs allow access for guest molecules to “touch” all the atoms of the skeleton. Therefore, CMPs represent an ideal platform for adsorbent materials. Moreover, the porosity of CMPs can be regulated by changing the size and geometric shape of the rigid monomers [33]. In other words, the desired structures of CMPs can be achieved through topology-directed design. This topology-directed strategy has been successfully applied to a series of PAFs to obtain low-crystalline or amorphous frameworks [32,33,34,35,36,37]. In some cases, preferential topological structures with regular pores have been achieved [38,39,40]. Therefore, the rational design of organic monomers under the guidance of topology chemistry enables organic porous frameworks to give predesigned structure and functionality. The symmetry of organic monomers affects pore structure and dimension, which are known to be determinative of the properties of materials [32]. Take iodine adsorption as an example, tuning pore sizes and pore structures of organic porous frameworks was found to play important roles in iodine uptake capacity [41,42,43,44,45].

**Figure 1:**
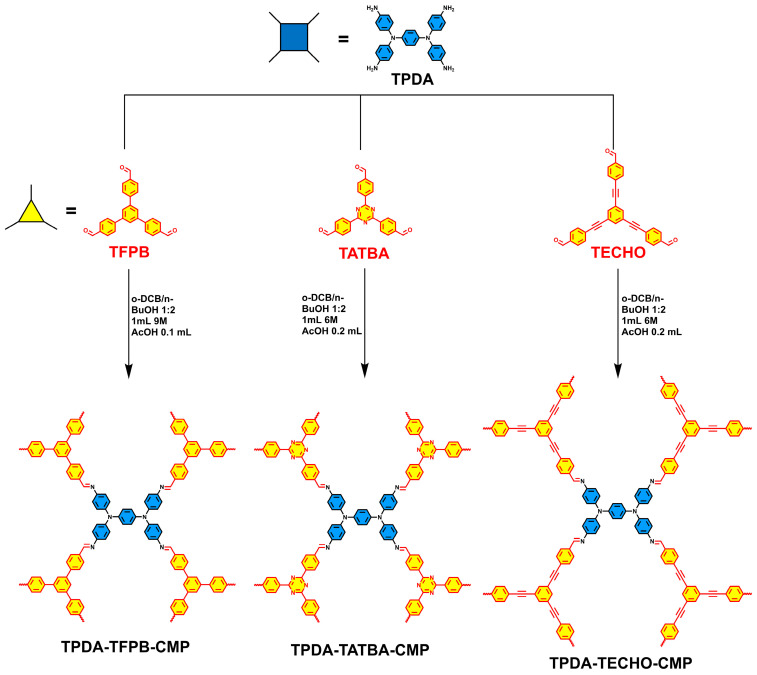
Synthesis routes for TPDA–TFPB CMP, TPDA–TATBA CMP, and TPDA–TECHO CMP.

Herein, we design and synthesize a series of new CMPs that were readily obtained through the condensation of a planar rectangular 4-connected N1,N1′-(1,4-phenylene)bis(N1-(4-aminophenyl)benzene-1,4-diamine) (TPDA) and triangular 3-connected 1,3,5-tris(4-formylphenyl)benzene (TFPB), 4,4′,4′′-(1,3,5-triazine-2,4,6-triyl)tris[benzaldehyde] (TATBA), and 4,4′,4′′-(1,3,5-benzenetriyltri-2,1-ethynediyl)tris[benzaldehyde] (TECHO) organic monomers, respectively (Figure 1). The combination of 4-connected and 3-connected monomers could present a preferential 3D network (see possible topological structures in Figure S1) although only amorphous structures of the CMPs are obtained in this work. The resultant TPDA–TFPB CMP, TPDA–TATBA CMP, and TPDA–TECHO CMP have moderate special surface areas (ranging from 262 to 426 m^2^ g^−1^) but show excellent volatile iodine adsorption capacities as high as 6.48, 6.25, and 6.37 g g^−1^, respectively. We consider that such high iodine adsorption capacities of the CMPs could be ascribed to their 3D topological structures, hierarchical pores, π-conjugated structure, uniform micromorphology, and high-density Lewis-base sites. In addition, the captured iodine within the porosities of CMPs can be rapidly released into methanol and the CMPs can be recycled many times without obvious loss in adsorption capability.

## 2. Results and Discussion

**Figure 2:**
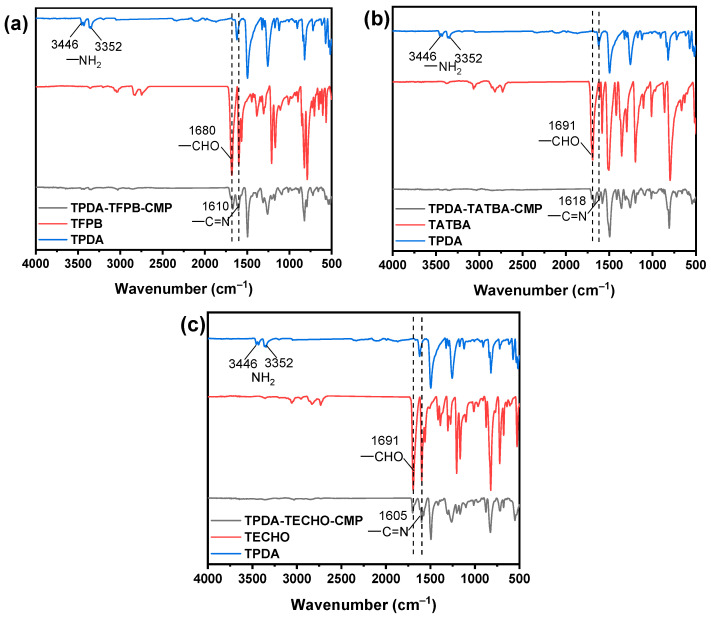
FT-IR spectra of TFPB, TPDA, and TPDA–TFPB CMP (**a**), TATBA, TPDA, and TPDA–TATBA CMP (**b**), and TECHO, TPDA, and TPDA–TECHO CMP (**c**).

Synthesis and characterization. The synthesis routes of the three CMPs, termed TPDA–TFPB CMP, TPDA–TATBA CMP, and TPDA–TECHO CMP, are shown in Figure 1. The synthesis details of the organic monomers and their corresponding ^1^H NMR spectra (Figures S2 and S3) [46,47] are given in supporting information (SI). The formation of the CMPs can be demonstrated by FT-IR spectra (Figure 2). It is clear that after condensation reactions, the stretching bands of -NH_2_ in TPDA at 3446 and 3352 cm^−1^ and the aldehyde stretching bands of 3-connected aromatic aldehydes (1680 cm^−1^ for TFPB; 1691 cm^−1^ for TATBA and TECHO) were remarkably decreased. Meanwhile, new peaks were observed (1610 cm^−1^ for TPDA–TFPB CMP, 1618 cm^−1^ for TPDA–TATBA CMP, and 1605 cm^−1^ for TPDA–TECHO CMP), which can be ascribed to the formation of imine groups. This reveals that the three CMPs were constructed through imine linkage. Note that the new peak of imine was superimposable on the signals of residual aldehyde groups, suggesting an incomplete condensation reaction. For this reason, the formation of imine linkages needs to be confirmed by solid-state ^13^C CP/MAS NMR spectroscopy (Figure S4). The peaks around 150–160 ppm can be attributed to the carbon atom of the imine linkages, while the peaks around 190–195 ppm can be assigned to the unreacted aldehyde groups. The incomplete condensation may be due to the highly branched connection of 4-connected and 3-connected monomers. It is also worth noting that the discrepancies between theoretical and experimental values in element analysis were observed in all POPs. The possible reason, we think, could be due to the organic residues that are not completely removed during the purification process or insufficient drying treatment that retains some organic solvents.

**Figure 3:**
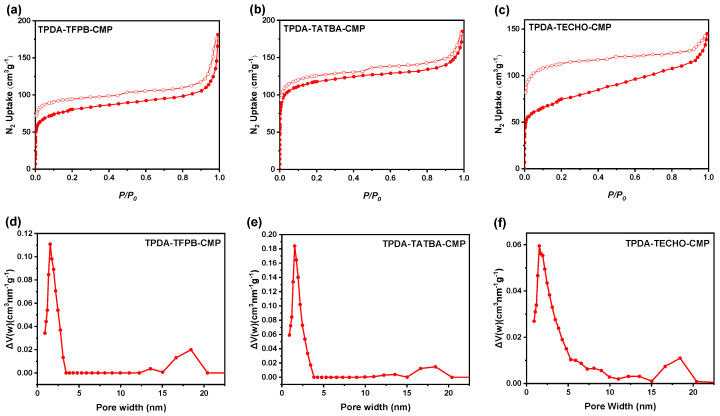
N_2_ adsorption and desorption isotherms of TPDA–TFPB CMP (**a**), TPDA–TATBA CMP (**b**), and TPDA–TECHO CMP (**c**), and their corresponding pore size distributions (**d**–**f**).

The porosity of the CMPs was characterized by nitrogen adsorption isotherm measurements at 77 K. It can be seen from Figure 3 that the CMPs exhibited combined Type I/IV sorption curves, suggesting the presence of micropores and mesopores. The N_2_ adsorption capacity of the CMPs increases sharply when the relative pressure, *P/P_0_*, is less than 0.1, indicating the existence of a large number of micropores in the three CMPs. When the *P/P_0_* ranges from 0.1 to 0.9, their N_2_ adsorption capacity increases slowly, with a hysteresis loop observed in the adsorption−desorption isotherms, which suggests the presence of mesoporous structures in the CMPs. Additionally, when the *P/P_0_* is in the range of 0.9 to 1.0, the N_2_ adsorption capabilities of TPDA–TFPB CMP, TPDA–TATBA CMP, and TPDA–TECHO CMP increase sharply again, indicating the coexistence of macropores in the three CMPs. The Brunauer−Emmett−Teller (BET) surface areas of TPDA–TFPB CMP, TPDA–TATBA CMP, and TPDA–TECHO CMP were calculated to be 284, 427, and 262 m^2^ g^−1^, and the corresponding pore volumes were estimated to be 0.26, 0.28, and 0.22 cm^3^ g^−1^, respectively. Based on the NLDFT, the average pore diameters of the three CMPs were estimated to be 2.5, 2.1, and 3.3 nm, respectively, revealing that the CMPs are mainly determined by their microporous structures.

**Figure 4:**
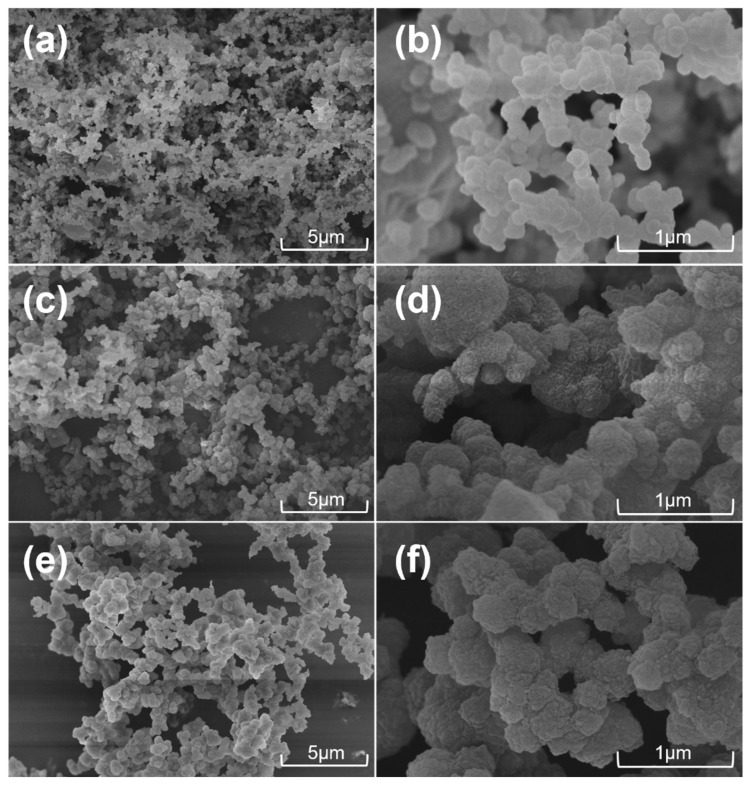
The FE-SEM images of TPDA–TFPB CMP (**a**,**b**), TPDA–TATBA CMP (**c**,**d**), and TPDA–TECHO CMP (**e**,**f**), as shown at different scale sizes.

The thermostability of the CMPs was investigated using TGA measurements (Figure S5). The three CMPs exhibited high decomposition temperatures of over 480 °C, revealing their good thermal stability. The micromorphology of the CMPs was characterized by FE-SEM observation. It is evident from Figure 4 that the CMPs are all composed of clusters of irregular particles. These particles are uniformly stacked, which is favorable for gas adsorption.

Iodine vapor capture by CMPs. Because of their uniform morphologies, high porosities, good thermostability, and 3D networks with π-conjugated structures, the CMPs can be good candidates for iodine vapor adsorption. The gravimetric method was used to investigate the volatile iodine capture capacity of CMPs as described in a previous report [16,21]. A certain amount of CMP adsorbent and excess iodine were separated and put in a closed bottle at 348 K and under ambient pressure. The changes in the weight of the CMP adsorbent were recorded over time, and thus the iodine adsorption capacity of the CMP at different time intervals was calculated (see details in the SI). The iodine vapor adsorption behaviors of the CMPs over time are shown inFigure 5. It is evident that iodine vapor can be quickly adsorbed in a short time, suggesting fast adsorption behavior and good adsorption capability. The hierarchical porous structures of the CMPs may lead to their fast adsorption behavior. The adsorption capability of the CMPs increased over time and reached the saturated adsorption capacity after about 60 h. The color of the CMP samples became deep/dark (Figure S6), suggesting a high degree of I_2_ adsorption. The saturated adsorption capacity of TPDA–TFPB CMP, TPDA–TATBA CMP, and TPDA–TECHO CMP was 6.48, 6.25, and 6.37 g g^−1^, respectively, demonstrating their excellent iodine adsorption capacities. Note that these values are higher than those in most of the reported materials (Table S1) [3,5,7,13,16,17,18,21,24,28,42,46,47,48,49,50,51,52,53,54,55,56,57,58,59,60,61,62,63,64,65,66,67,68,69,70,71,72,73,74,75,76,77,78,79,80,81,82,83,84,85,86,87].

The 4-connected and 3-connected building units are both planar π-conjugated molecular structures; however, the combination of 4-connected and 3-connected building units produces 3D extended networks [88,89]. Despite the amorphous structure of the CMPs obtained in this work (Figure S7), it can be predicted that their networks will grow preferentially along the predesigned topological structure to the present 3D skeletons. Actually, some PAFs constructed by irreversible covalent bonds have shown low crystalline structures with ordered porosity [34,35,36,37]. Therefore, we believe that the 3D extended networks with the accessible π-conjugated structure of the CMPs could lead to their ultrahigh iodine adsorption capacity. In addition, the high density of electron-donating imine linkages throughout the skeletons of CMPs also contributes to their adsorption capacity [16,21]. After I_2_ saturation adsorption, no diffraction peaks attributable to I_2_ crystals were observed in all I_2_≅CMPs (Figure S7), suggesting that no crystalline iodine was deposited on the surface of CMPs. The retention capacity of iodine is also an important parameter for characterizing iodine adsorbent performance. As shown in Figure S5, the iodine-loaded CMPs (designated as I_2_≅CMP) showed a mass loss of iodine at about 100 °C, revealing their good retention capacities. The I_2_ adsorption capabilities of the CMPs in n-hexane solvent were also investigated using UV-vis spectra (Figures S8 and S9). The adsorption capabilities of TPDA–TFPB CMP, TPDA–TATBA CMP, and TPDA–TECHO CMP were 200, 157, and 160 mg g^–1^, respectively, when the concentration of I_2_ in n-hexane was 100 mg L^–1^. Their corresponding adsorption capabilities were 542, 547, and 488 mg g^–1^ when the concentration of I_2_ in n-hexane was 300 mg L^–1^, and 874, 850, and 784 mg g^–1^ when the concentration of I_2_ in n-hexane was 500 mg L^–1^. Clearly, the CMPs also exhibited good I_2_ adsorption capabilities in n-hexane. 

**Figure 6:**
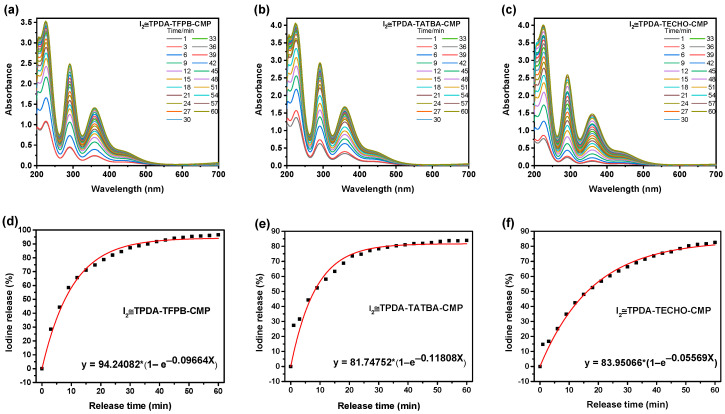
The behavior of iodine release from the I_2_≅TPDA–TFPB CMP (**a**,**d**), I_2_≅TPDA–TATBA CMP (**b**,**e**), and I_2_≅TPDA–TECHO CMP (**c**,**f**) into methanol with time.

Behavior of iodine release from CMPs and their recyclability. The behavior of iodine release from CMPs was also investigated. Iodine release into methanol was monitored by UV−vis adsorption spectroscopy at room temperature (Figure S10).Figure 6 shows the behavior of iodine release from the iodine-saturated CMPs. It can be seen that the release ratio of the CMPs can reach 80 wt%, although TPDA–TFPB CMP demonstrated a release ratio of nearly 100 wt%. The release curves were fitted using Origin software version 10.1 (The simulated equations are inserted in Figure 6). It can be seen that the concentration of iodine released into methanol increased exponentially with time, suggesting an ultrafast release capability of the CMPs, which is different from the MOFs that released iodine into a solvent in a linear fashion [50,51]. However, the release rates of the I_2_-loaded CMPs (I_2_≅CMPs) are slower than those of 2D or 3D COFs [16,43]. The reason is that COFs have ordered one-dimensional (1D) open channel structures favoring the ultrarapid release of iodine molecules. The recyclability of the CMPs was also investigated (Figure S11). The iodine adsorbed by I_2_≅TPDA–TFPB CMP, I_2_≅TPDA–TATBA CMP, and I_2_≅TPDA–TECHO CMP was completely removed into methanol by Soxhlet extraction, and the empty CMPs were dried and then reused to capture iodine vapor. The same procedure was repeated several times. It is evident that the iodine adsorption capabilities of the three CMPs decreased slightly after five cycles; however, more than 80% adsorption capacity was still maintained, suggesting their good recyclability.

**Figure 7:**
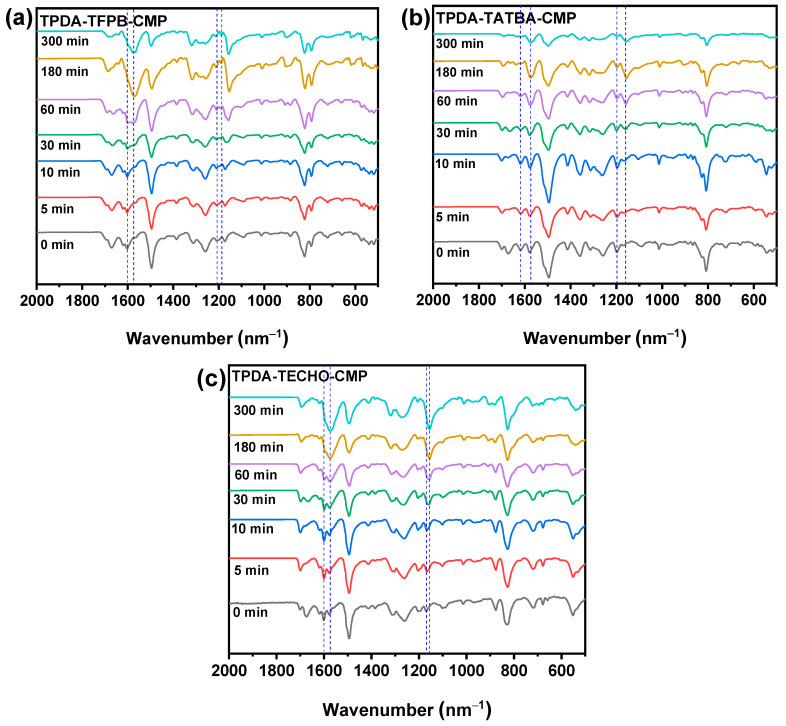
Effect of adsorbed iodine on the FT-IR spectra of TPDA-TFPB-CMP (**a**), TPDA-TATBA-CMP (**b**), and TPDA-TECHO-CMP (**c**).

Investigation of adsorption mechanism. To analyze the interaction between the adsorbed iodine and the skeleton of the CMPs, FT-IR measurements were used to investigate the effect of iodine on the FT-IR spectrum (Figure 7). It is clear that the FT-IR spectrum changed with adsorption time. Take TPDA–TFPB CMP as an example; the stretching vibration of the imine linkages at 1610 cm^–1^ shifted to 1580 cm^–1^ in the first 10 min, suggesting that there are strong interactions between the iodine and the imine groups. The Lewis-base imines are the preferred adsorption sites because of the Lewis-acidic nature of iodine [16]. This peak of the imine disappeared gradually, and a new peak concomitantly appeared with adsorption time, which indicates that the iodine was chemically adsorbed by the imine linkages and charge transfer complexes formed between the iodine and the imine linkages [44]. Likewise, these changes were also observed in the stretching vibration of -C-N- of tertiary ammonia at 1200 cm^–1^ in the TPDA group of the CMPs, suggesting that the tertiary ammonia groups are also strong chemical adsorption sites for iodine. These changes in imine and tertiary ammonia groups were also observed in TPDA–TATBA CMP and TPDA–TECHO CMP. The strong chemical adsorption can be considered the main reason that the CMPs exhibited ultrahigh iodine adsorption capabilities. In addition, the 3D network structures with accessible hierarchical pores, uniform micromorphology, and high-density Lewis-base imine linkages of the CMPs may synergistically contribute to their excellent iodine adsorption performance.

**Figure 8:**
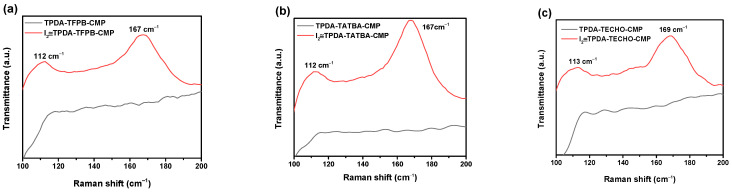
Effects of adsorbed iodine on the Raman spectra of TPDA–TFPB CMP (**a**), TPDA–TATBA CMP (**b**), and TPDA–TECHO CMP (**c**).

The Raman spectrum is another powerful tool for characterizing the formation of polyiodide anions. After the adsorption of iodine, two clear bands, at 167 and 112 cm^−1^, for I_2_≅TPDA–TFPB CMP and I_2_≅TPDA–TATBA CMP, and at 169 and 113 cm^−1^ for I_2_≅TPDA–TECHO CMP were observed (Figure 8). The bands at low wavenumbers (112 and 113 cm^−1^) are due to the symmetric stretching vibration of I_3_^–^ ions, and the bands at high wavenumbers (167 and 169 cm^−1^) are associated with the stretching vibration of polyiodide I_5_^–^ [16,21,53]. These results confirm the formation of charge transfer complexes between the captured iodine and the imines, as well as the tertiary ammonia groups of the CMPs.

## 3. Experimental Section

Materials. Solvents were purchased from Tansoole and used without any purification. The organic monomers N1,N1′-(1,4-phenylene)bis(N1-(4-aminophenyl)benzene-1,4-diamine) (TPDA) and 4,4′,4′′-(1,3,5-benzenetriyltri-2,1-ethynediyl)tris[benzaldehyde] (TECHO) were purchased from Bide Pharmatech Ltd., Shanghai, China, while 1,3,5-tris(4-formylphenyl)benzene (TFPB) and 4,4′,4′′-(1,3,5-triazine-2,4,6-triyl)tris[benzaldehyde] (TATBA) were synthesized according to documented procedures [46,90].

Synthesis of TPDA–TFPB CMP. To a 5 mL Pyrex tube was added TPDA (12.8 mg, 0.027 mmol), TFPB (15.6 mg, 0.04 mmol), and a mixture of o-dichlorobenzene (0.5 mL) and n-butanol (0.5 mL). This was followed by exposure to ultrasound for 15 min. After that, 0.1 mL acetic acid (9 M) was added to the tube, which was then flash-frozen through liquid nitrogen and degassed via five freeze–pump–thaw cycles. The tube was sealed with a flame and then heated at 120 °C for 3 days. After the reaction was complete, the resultant precipitate was immersed in dried DMF for 2 days and further purified by Soxhlet extraction with anhydrous tetrahydrofuran/DMF (1:2, *v*/*v*) for 2 days. The solid was dried under vacuum at 120 °C to give yellow TPDA–TFPB CMP (80% yield). Elemental analysis (%) of TPDA–TFPB CMP was conducted. We expected C, 86.06; H, 4.81; N 9.12 and found C, 81.87; H, 5.01; N 9.63.

Synthesis of TPDA–TATBA CMP. To a 5 mL Pyrex tube was added TPDA (12.8 mg, 0.027 mmol), TATBA (14.2 mg, 0.036 mmol), and a mixture of o-dichlorobenzene (0.5 mL) and n-butanol (0.5 mL). This was followed by exposure to ultrasound for 15 min. After that, 0.2 mL acetic acid (6 M) was added to the tube, which was then flash-frozen through liquid nitrogen and degassed via five freeze–pump–thaw cycles. The tube was sealed with a flame and then heated at 120 °C for 3 days. After the reaction was complete, the resultant precipitate was immersed in dried DMF for 2 days and further purified by Soxhlet extraction with anhydrous tetrahydrofuran/DMF (1:2, *v*/*v*) for 2 days. The solid was dried under vacuum at 120 °C to give a red TPDA–TATBA CMP (81% yield). Elemental analysis (%) of TPDA–TATBA CMP was conducted. We expected C, 80.51; H, 4.33; N 15.15 and found C, 75.66; H, 4.49; N 14.56.

Synthesis of TPDA–TECHO CMP. The procedure for synthesizing TPDA–TECHO CMP was similar to that of TPDA–TATBA CMP, but with TPDA (12.8 mg, 0.027 mmol) and TECHO (16.7 mg, 0.036 mmol) used as organic monomers instead. This approach gave a yellowish-red powder (83% yield). Elemental analysis (%) of TPDA–TECHO CMP was conducted. We expected C, 87.38; H, 4.36; N 8.26 and found C, 83.06; H, 4.53; N 8.16.

## 4. Methods

Fourier transform infrared (FT-IR) spectra were recorded with a Jasco FT/IR-6800 spectrometer (Japan) in the range of 4000–400 cm^−1^. The spectral signals are presented in wavenumbers (cm^−1^). The Powder X-ray diffraction (PXRD) patterns were collected on a Rigaku X-ray diffractometer (MiniFlex600) using Cu-Kα radiation (λ = 1.542 Å). The patterns were recorded in the 2θ range from 2° to 30° with a step size of 0.02° and exposure time of 10° min^–1^. The porous nature of the samples was determined by N_2_ adsorption/desorption isotherms at 77 K using Quantachrome Autosorb-iQ (Shanghai, China). Before testing, the samples were dried under vacuum for 12 h at 120 °C. Pore size distribution was calculated using nitrogen adsorption data based on the nonlocal density functional theory (NLDFT) approach. Thermogravimetric analyses (TGA) were carried out on a Thermobalance TGA Q600 thermal gravimetric analyzer under a nitrogen atmosphere. The samples were heated at a rate of 10 °C min^−1^ from room temperature to 700 °C. Field-emission scanning electron microscopy (FE-SEM) measurements were performed on a Hitachi Regulus8100. Ultraviolet visible infrared spectrophotometer (UV-vis) spectra were recorded with a Jasco V-770 spectrophotometer in the range of 200–700 nm. ^1^H NMR and ^13^C NMR spectra were recorded on a 400 M NMR spectrometer (Bruker AVANCE NEO 400 MHz NMR spectrometer). ^13^C cross-polarization magic-angle spinning nuclear magnetic resonance (^13^C CP/MAS NMR) spectroscopy was recorded on Bruker AVANCE III spectrometer (600 MHz). Raman spectrum was recorded on an InVia Qontor spectrometer with an excitation wavelength of 532 nm at room temperature.

## 5. Conclusions

In summary, three novel conjugated microporous polymers (CMPs) were designed and synthesized under the guidance of topology. A planar rectangular 4-connected organic monomer (TPDA) was used to construct preferred three-dimensional (3D) networks with triangular 3-connected organic monomers (TFPB, TATBA, and TECHO) to produce TPDA–TFPB CMP, TPDA–TATBA CMP, and TPDA–TECHO CMP. The high porosity, good thermostability, uniform micromorphology, and π-conjugated structures of the CMPs encouraged us to investigate their iodine adsorption behaviors. The resultant CMPs exhibited ultrahigh iodine adsorption capacities of 6.48, 6.25, and 6.37 g g^−1^, respectively, at 348 K and ambient pressure. FT-IR and Raman spectra were used to analyze the adsorption mechanism of iodine. The strong chemical adsorption between the iodine and the imine/tertiary ammonia of the CMPs may contribute to the ultrahigh iodine adsorption capacities. In addition, the 3D network structures with accessible hierarchical pores, uniform micromorphology, and high-density Lewis-base sites (imine and tertiary ammonia of TPDA) could also synergistically lead to their excellent iodine adsorption performance. This research gives guidance for the design of novel CMPs to cope with nuclear pollution in the field of nuclear power.

## Figures and Tables

**Figure 5:**
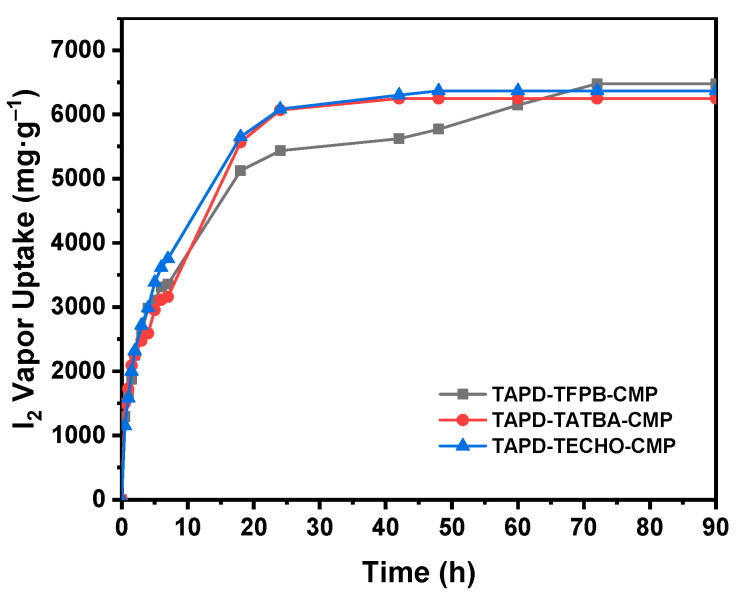
Iodine vapor adsorption curves of TPDA–TFPB CMP, TPDA–TATBA CMP, and TPDA–TECHO CMP at 348 K under ambient pressure.

## Data Availability

The authors confirm that most of the data supporting the findings of this study are available within the article and its Supplementary Materials. Raw data are available from the corresponding author (Y.G.) on request.

## Supplementary Materials

The following supporting information can be downloaded at: https://www.mdpi.com/article/10.3390/molecules29102242/s1, Figure S1. The possible 3D topological structures through the combination of 4-connected and 3-connected monomers; Figure S2. ^1^H NMR spectrum of TFPB; Figure S3. ^1^H NMR spectrum of TAPBA; Figure S4. ^13^C CP/MAS NMR spectra of TPDA-TFPB-CMP, TPDA-TATBA-CMP, and TPDA-TECHO-CMP; Figure S5. TGA curves of TPDA-TFPB-CMP and I_2_≅TPDA-TFPB-CMP (a), TPDA-TATBA-CMP and I_2_≅TPDA-TATBA-CMP (b), and TPDA-TECHO-CMP and I_2_≅TPDA-TECHO-CMP (c); Figure S6. The color change of TPDA-TFPB-CMP (left), TPDA-TATBA-CMP (middle), and TPDA-TECHO-CMP (right) before and after I_2_ adsorption; Figure S7. PXRD patterns of TPDA-TFPB-CMP and I_2_≅TPDA-TFPB-CMP (a) TPDA-TATBA-CMP and I_2_≅TPDA-TATBA-CMP (b), and TPDA-TECHO-CMP and I_2_≅TPDA-TECHO-CMP (c); Table S1. Comparison of representatively reported adsorbents with our work for iodine vapor adsorption under atmospheric pressure; Figure S8. UV-vis spectra of n-hexane standard solutions of iodine with different concentration (a) and the corresponding calibration curve of absorbance versus iodine concentration established from UV-vis spectra (b) as shown in (a); Figure S9. The I_2_ adsorption behavior of the CMPs in n-hexane. The concentration of iodine in n-hexane solution is 100 mg L^–1^, 300 mg L^–1^, and 500 mg L^–1^, respectively; Figure S10. UV-vis spectra of methanol standard solutions of iodine with different concentration (a) and the corresponding calibration curve of absorbance versus iodine concentration established from UV-vis spectra (b) as shown in (a); Figure S11. Recyclability of TPDA-TFPB-CMP, TPDA-TATBA-CMP, and TPDA-TECHO-CMP in iodine uptake.
