# Supplementary material for: Highly Efficient Capture of Volatile Iodine by Conjugated Microporous Polymers Constructed Using Planar 3- and 4-Connected Organic Monomers
Source: Molecules. 2024 May 10;29(10):2242. doi: 10.3390/molecules29102242 (PMC11124010; doi:10.3390/molecules29102242)
Supplement: Supplementary file 1 [file molecules-29-02242-s001.zip › molecules-2964363-supplementary.pdf]

molecules

MDPI

Supplementary Information

Highly Efficient Capture of Volatile Iodine by Conjugated Microporous Polymers Constructed using Planar 3- and 4-Connected Organic Monomers

Chaohui Li [1,†], Qianqian Yan [1,†], Huanjun Xu [2], Siyu Luo [1], Hui Hu [1,*], Shenglin Wang [1,*], Xiaofang Su [1], Songtao Xiao [3] and Yanan Gao [1]

[1] key Laboratory of Ministry of Education for Advanced Materials in Tropical Island Resources, Hainan University, No 58, Renmin Avenue, Haikou 570228, China
[2] School of Science, Qiongtai Normal University, Haikou 571127, China
[3] China Institute of Atomic Energy, Beijing 102413, China
* Correspondence: hhu@hainanu.edu.cn (H.H); wangshenglin@hainanu.edu.cn (S.W)
†C. L and Q. Y contributed equally to this work.

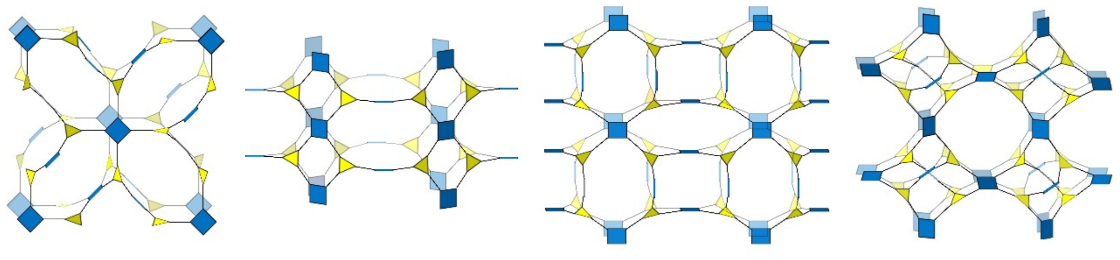

Figure S1. The possible 3D topological structures through the combination of 4-connected and 3-connected monomers.

Synthesis of 1,3,5-tris(4-formylphenyl)benzene (TFPB)

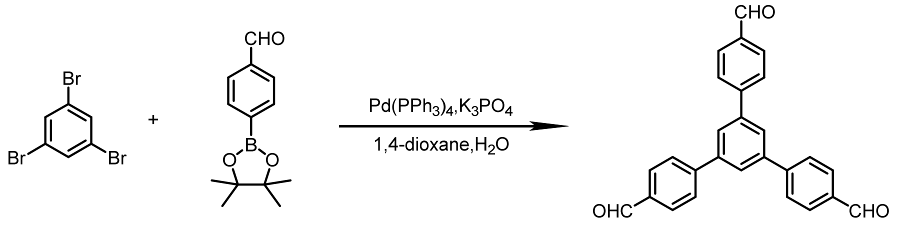

TFPB was synthesized according to the reported method in the literature.[1] Typically, a mixture of 4-(4,4,5,5-tetramethyl-1,3,2-dioxaborolan-2-yl)benzaldehyde (4.64 g, 20 mmol), 1,3,5-tribromobenzene (1.57 g, 5 mmol), $Pd(PPh_3)_4$ (288.9 mg, 0.25 mmol), potassium phosphate (7.43 g, 35 mmol), 1,4-dioxane (80.0 mL) and distilled water (8.0 mL) was stirred at 90 °C under a N_2 atmosphere for 24 h. After cooling down to room temperature, the solvent was removed under vacuum and the residue was purified by flash column chromatography (petroleum ether/ethyl acetate/dichloromethane (10/1; 4/1); silica gel, 300-400 mesh) to give TFPB as a white solid (1.7 g, 87%). ^1H NMR (400 MHz, $CDCl_3$): δ 10.07 (s, 3H), 8.03 (d, J = 8.2 Hz, 6H), 7.91 (s, 3H), 7.88 (d, J = 8.2 Hz, 6H). The ^1H NMR spectrum of TFPB is shown in Figure S2.

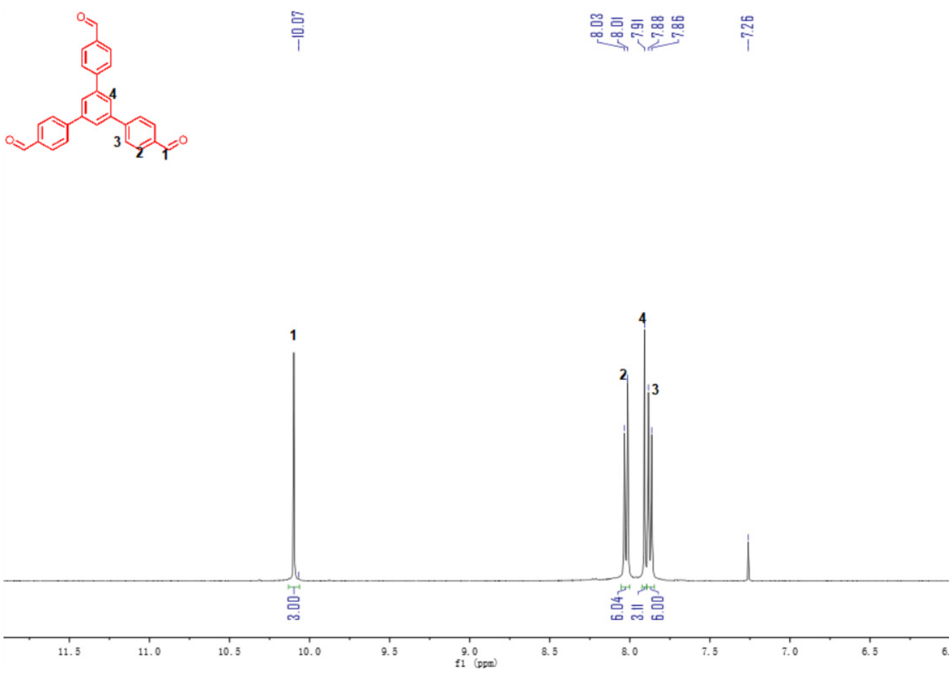

Figure S2. ^{1}H NMR spectrum of TFPB.

Synthesis of 4,4′,4′′-(1,3,5-triazine-2,4,6-triyl)tris[benzaldehyde] (TATBA)

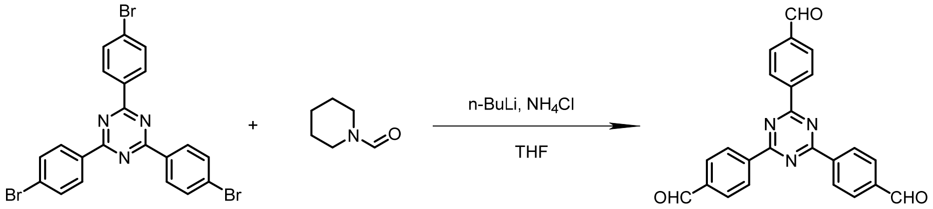

TAPBA was synthesized according to the reported method in the literature.[2] To a suspension of 2,4,6-tris(4-bromophenyl)-1,3,5-triazine (0.50 g, 0.92 mmol, 1.0 eq.) in THF (50.0 mL), n-BuLi (2.5 M in hexane, 1.2 mL, 3.0 mmol, 3.3 eq.) was added dropwise at -78 °C. The mixture was stirred at -78 °C for 90 min, then 1-formylpiperidine (0.34 mL, 3.0 mmol, 3.3 eq.) was added dropwise at -78 °C. The mixture was stirred at -78 °C for 30 min and allowed to warm to room temperature. Aqueous concentrated NH_4Cl solution (1.0 mL) was added and the solvent was removed under reduced pressure. The residue was suspended in a mixture of EtOH (3.0 mL) and water (3.0 mL). Suction filtration of the suspension and washing with water (10.0 mL) and EtOH (10.0 mL) afforded TAPBA (0.35 g, 0.91 mmol, 97 %) as an off-white solid. ^{1}H NMR (400 MHz, $CDCl_3$): δ = 10.21 (s, 3H, H-1), 8.95 (d, J = 8.2 Hz, 6H, H-6), 8.16–8.11 (m, 6H, H-5) ppm.

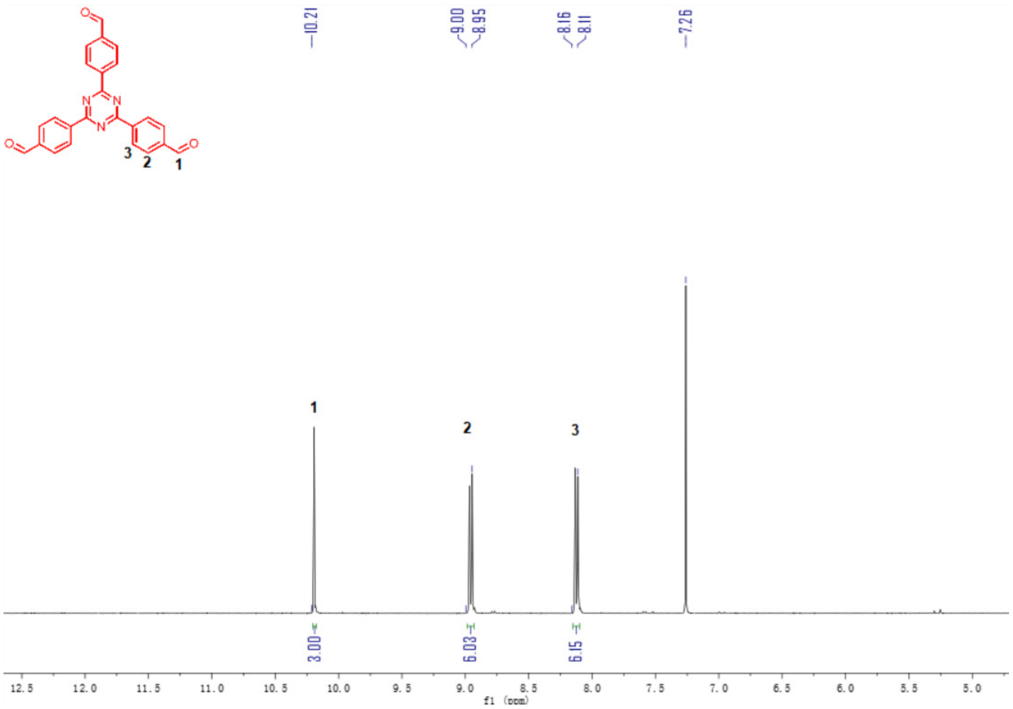

Figure S3. ^{1}H NMR spectrum of TAPBA.

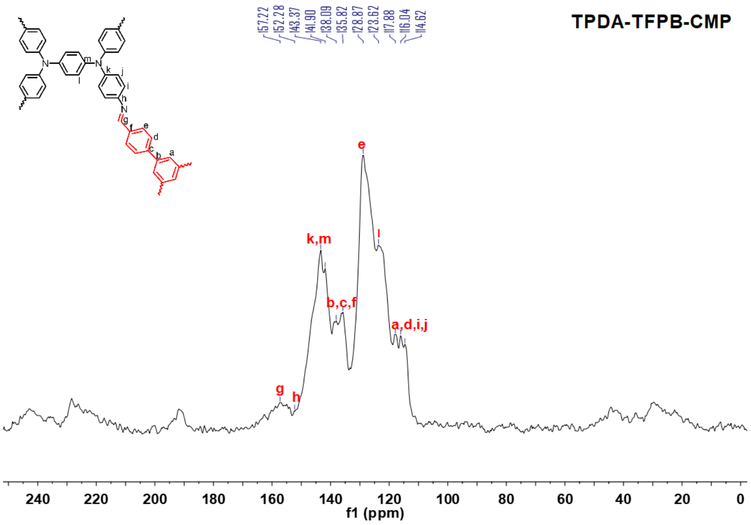

(**a**)

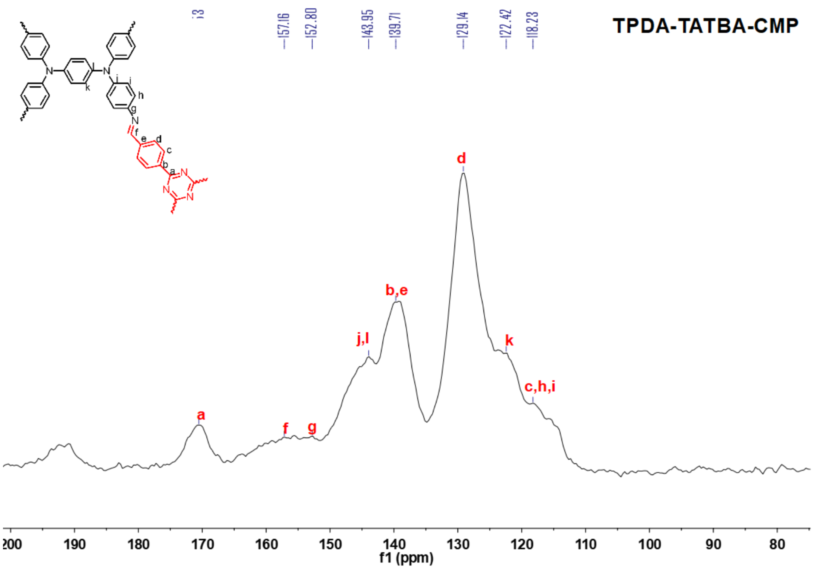

(**b**)

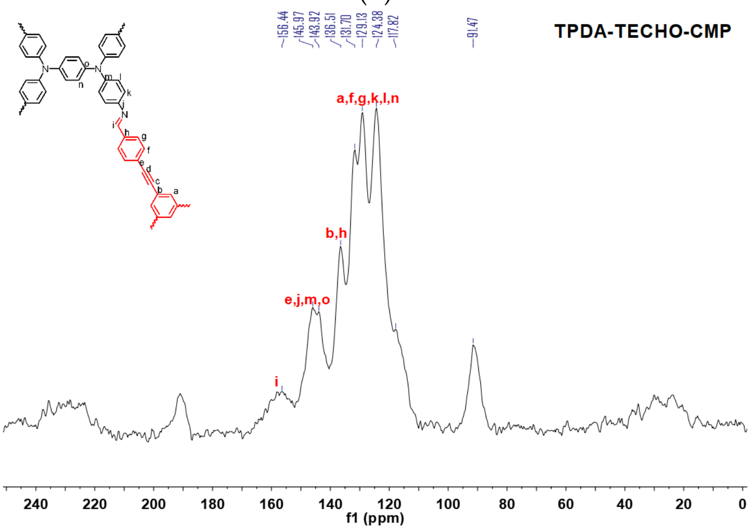

(**c**)

Figure S4. ^{13}C CP/MAS NMR spectra of TPDA-TFPB-CMP (a), TPDA-TATBA-CMP (b), and TPDA-TECHO-CMP (c).

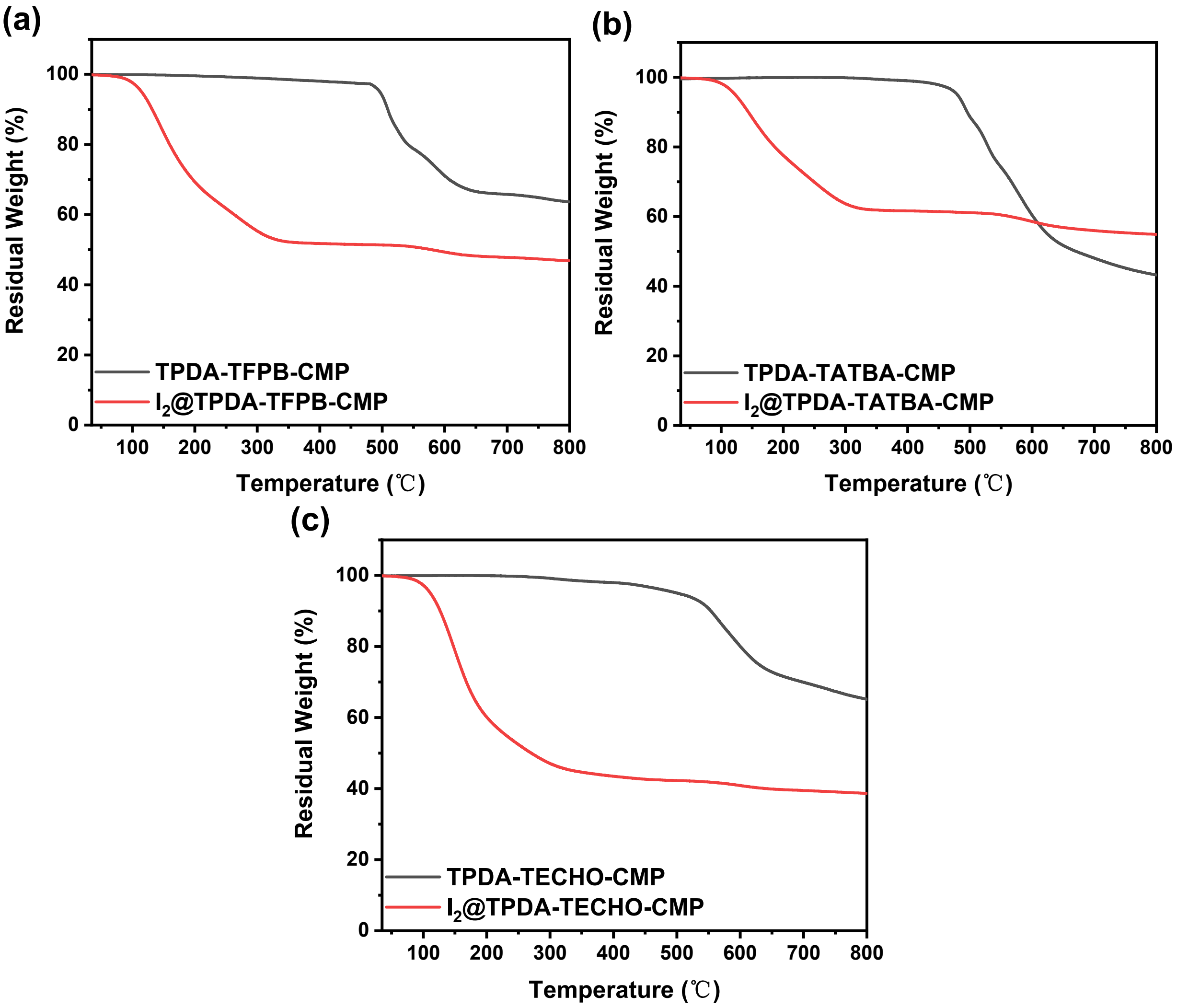

Figure S5. TGA curves of TPDA-TFPB-CMP and I_2@TPDA-TFPB-CMP (a), TPDA-TATBA-CMP and I_2@TPDA-TATBA-CMP (b), and TPDA-TECHO-CMP and I_2@TPDA-TECHO-CMP (c).

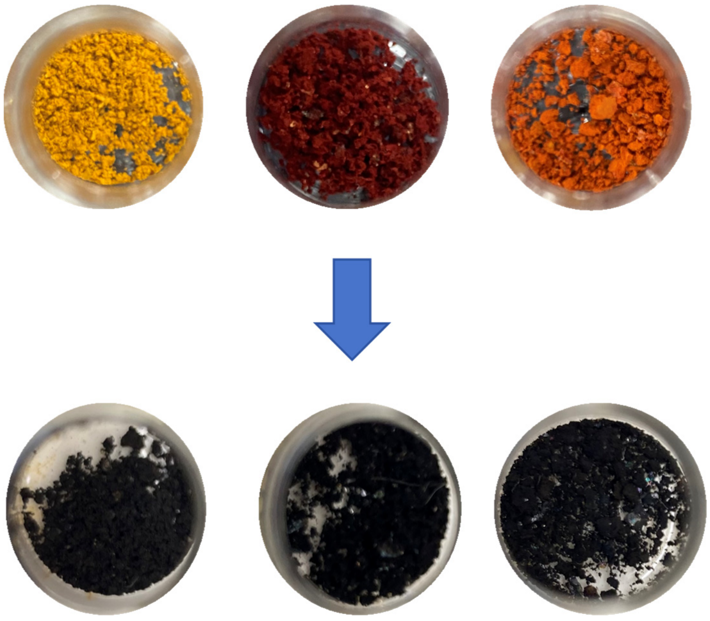

Figure S6. The color change of TPDA-TFPB-CMP (left), TPDA-TATBA-CMP (middle), and TPDA-TECHO-CMP (right) before and after I_2 adsorption.

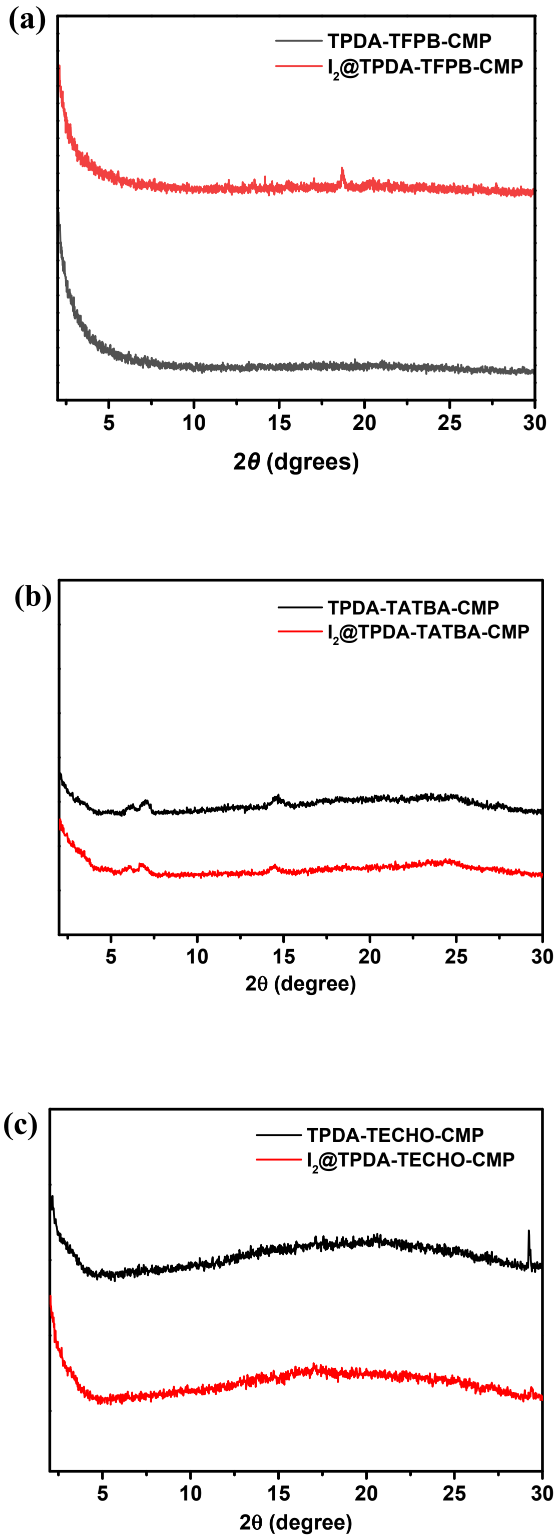

Figure S7. PXRD patterns of TPDA-TFPB-CMP and I_2≅TPDA-TFPB-CMP (a) TPDA-TATBA-CMP and I_2≅ΤΠΔΑ-TATBA-CMP (b), and TPDA-TECHO-CMP and I_2≅TPDA-TECHO-CMP (c).

Iodine vapor adsorption experimental procedure.

5.0 mg of CMP adsorbent was loaded in a small open vial and transferred into a big chamber. Excess of iodine powder was put at the bottom of the chamber, that was sealed tightly and moved to a convection oven (348 K) for iodine vapor adsorption experiment

under ambient pressure. The weight of the vial that loaded CMPs was recorded at different exposure times and the adsorption curves of the samples were thus plotted.

The iodine adsorption capacity of CMPs was evaluated according to the following equation:

$$\alpha = \frac{(m_2 - m_1)}{m_1} \quad (1)$$

where α is the iodine vapor uptake capacity, and m_1 and m_2 represent the weight of CMP sample before and after the iodine adsorption.

Table S1. Comparison of representatively reported adsorbents with our work for iodine vapor adsorption under atmospheric pressure.

Adsorbents	S_{BET} (m^2 g^{-1})	Pore volume (cm^3 g^{-1})	Temperature (°C)	Iodine adsorption capability (g g^{-1})	Ref.
iCOFs-AB-50	1390	1.21	75	10.21	(3)
iCOFs-AB-33	1580	1.39	75	9.00	(3)
iCOFs-AB-67	1253	0.80	75	8.51	(3)
JUC-561	2359	1.92	75	8.19	(4)
TPDA-TFPB-CMP	284	0.26	75	6.48	This work
TPDA-TATBA-CMP	427	0.28	75	6.37	This work
QTD-COF-V	n.a.	n.a.	75	6.29	(5)
TPB-DMTP	1927	1.28	77	6.26	(6)
TPDA-TECHO-CMP	262	0.22	75	6.25	This work
TJNU-201	2510	n.a.	77	5.625	(7)
TPT-BD-COF	109	0.3	75	5.43	(8)
TTA-TTB COF	1733	1.01	77	4.95	(6)
TTPPA	512.39	0.13	77	4.90	(9)
TJNU-202	714	n.a	77	4.82	(7)
SIOC-COF-7	618	0.41	75	4.81	(10)
TPE-TPDA-CMP	634	0.63	75	4.68	(11)
ETTA-PyTTA-COF	1519	0.82	75	4.6	(12)
TPT-DHBD50 COF	124	0.19	75	4.3	(8)
TPT-DHBD75 COF	157	0.19	75	4.12	(8)
NDB-H	116.9	0.13	75	4.43	(13)
NDB-S	56.5	0.11	75	4.25	(13)
POP-T	18.3	0.027	75	3.94	(14)
POP-2	41	n.a.	80	3.82	(15)
CalPOF-2	154	n.a.	75	4.06	(16)
COF-320	2400	0.81	75	4.0	(17)
Meso-COF-3	982	0.84	75	4.0	(17)
TPT-DHBD-COF	297	0.54	75	3.88	(8)
CTF-CTTD-500	1334	1.40	78	3.87	(18)
KOH-AC	1973	1.15	77	3.76	(19)
TPE-TAPP-CMP	454.1	0.58	75	3.67	(11)
CTF-CTTD-400	1684	1.44	78	3.57	(18)
CalPOF-3	91	n.a.	75	3.53	(16)
Micro-COF-2	1056	0.71	75	3.5	(17)
COF-300	1360	0.72	75	3.5	(17)
HCMP-3	82	0.08	85	3.36	(20)
Meso-COF-4	926	1.01	75	3.3	(17)
TPE-PyTTA-CMP	669.9	0.4	75	3.10	(11)
MIL-101	3134	1.52	77	3.02	(21)
AzoPPN	400	0.68	77	2.9	(22)
Micro-COF-1	816	0.59	75	2.9	(23)
PAN3	194	0.15	75	2.81	(23)

PAF-24	136	n.a.	75	2.76	(24)
TTA-TFB COF	1163	0.55	75	2.76	(6)
PAF-23	82	n.a.	75	2.71	(24)
PAF-25	262	n.a.	75	2.60	(24)
Cg-5C	1200	2.3	25	2.39	(25)
Azo-Trip	510.4	0.47	80	2.38	(26)
TPT-TAPB-COF	957	0.57	78	2.25	(27)
NiMoS chalcogels	490	n.a.	60	2.25	(28)
BDP-CPP-2	n.a.	n.a.	75	2.23	(29)
SCMP-2	n.a.	n.a.	60	2.22	(30)
TPT-Azine-COF	1020	0.65	78	2.19	(27)
MOF-808	1930	0.82	80	2.18	(31)
CMP-E1	n.a.	n.a.	75	2.15	(24)
CMPN-3	1368	2.36	75	2.08	(32)
NiP-CMP	2630	2.288	80	2.02	(33)
NTP	1067	n.a.	75	1.80	(34)
Cu-BTC	n.a.	n.a.	75	1.75	(35)
PAF-21	n.a	n.a	75	1.52	(24)
ZIF-8	1837	n.a	80	1.25	(36)
Ni(44dba)$_2$	n.a	n.a	RT	1.1	(37)
POP-E	37	0.058	75	3.49	(14)
POP-P	27.8	0.037	75	3.27	(14)
BDP-CPP-1	635	0.78	75	2.83	(38)
BDP-CPP-2	235	0.18	75	2.23	(38)
Por-Py-CMP	1014	0.81	77	1.30	(39)
CMPN-2	339	0.39	75	1.10	(40)
CMPN-1	230	0.14	75	0.97	(40)
JUC-Z2	2081	1.45	60	0.80	(41)
PPy	6	n.a.	80	0.63	(42)
Cg-5P	957	1.62	25	0.87	(43)
CF/COF monolith	166	n.a.	80	0.82	(43)
HKUST-1	n.a.	n.a.	75	0.636	(44)
Polyurethane (PU1)	n.a.	n.a.	70	0.565	(45)
$[Zn_3(DL\text{-}lac)_2(pybz)_2]_3(I_2)$	918.5	n.a.	25	0.497	(46)
3D Cd (II)-triazole MOF	n.a.	n.a.	25	0.46	(47)
$\{[Cu_6(pybz)_8(OH)_2]\ I_5^-I_7^-\}_n$	n.a.	n.a.	140	0.432	(48)
CC3	n.a.	n.a.	20	0.364	(49)
$[Fe_3(HCOO)_6]\ (I_2)_{0.84}$	385	n.a.	25	0.328	(50)
Ag-MOR	n.a.	n.a.	75	0.28	(51)
Ag@Zeolite Mordenitres	n.a.	n.a.	95	0.275	(52)
Ag@Mon-POF	1230	1.46	70	0.25	(53)
Macroreticular resins	n.a.	n.a.	<50	0.2-1.0	(54)
AgX-silverexchanged faujasite	n.a.	n.a.	150	0.08-0.20	(54)
AgZ-silver exchanged mordenite	n.a.	n.a.	150	0.17	(54)
$[Zn(C_8H_8O_8)]\cdot 2H_2O$	n.a.	n.a.	19	0.166	(55)
AC-6120-silver impregnated silica gel	n.a.	n.a.	130	0.135	(55)
AgA-silver impregnated alumina	n.a.	n.a.	150	0.100-0.235	(54)

n.a: no data was given.

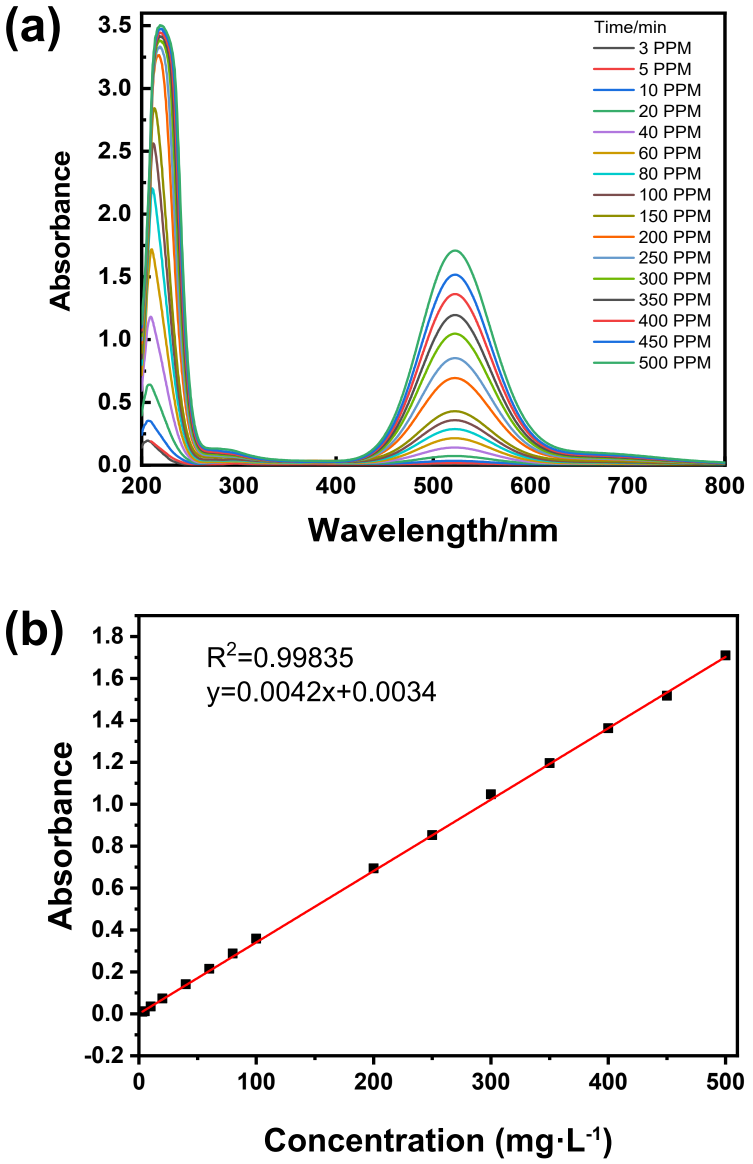

Figure S8. UV-vis spectra of n-hexane standard solutions of iodine with different concentration (a) and the corresponding calibration curve of absorbance versus iodine concentration established from UV-vis spectra (b) as shown in (a).

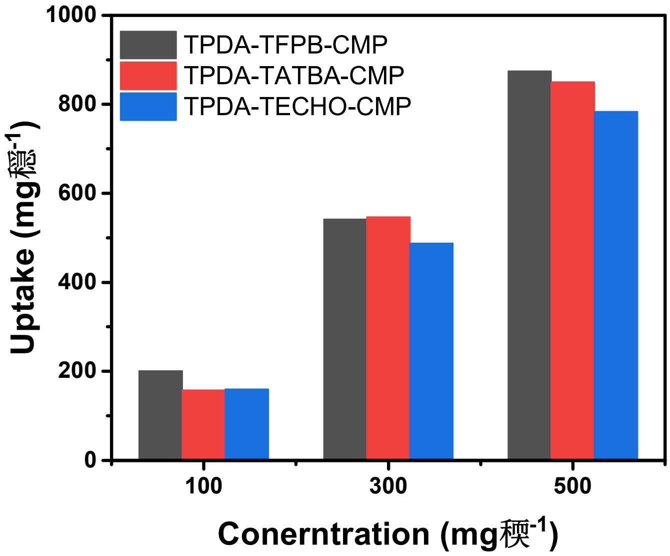

Figure S9. The I_2 adsorption behavior of the CMPs in n-hexane. The concentration of iodine in n-hexane solution is 100 mg L^{-1}, 300 mg L^{-1}, and 500 mg L^{-1}, respectively.

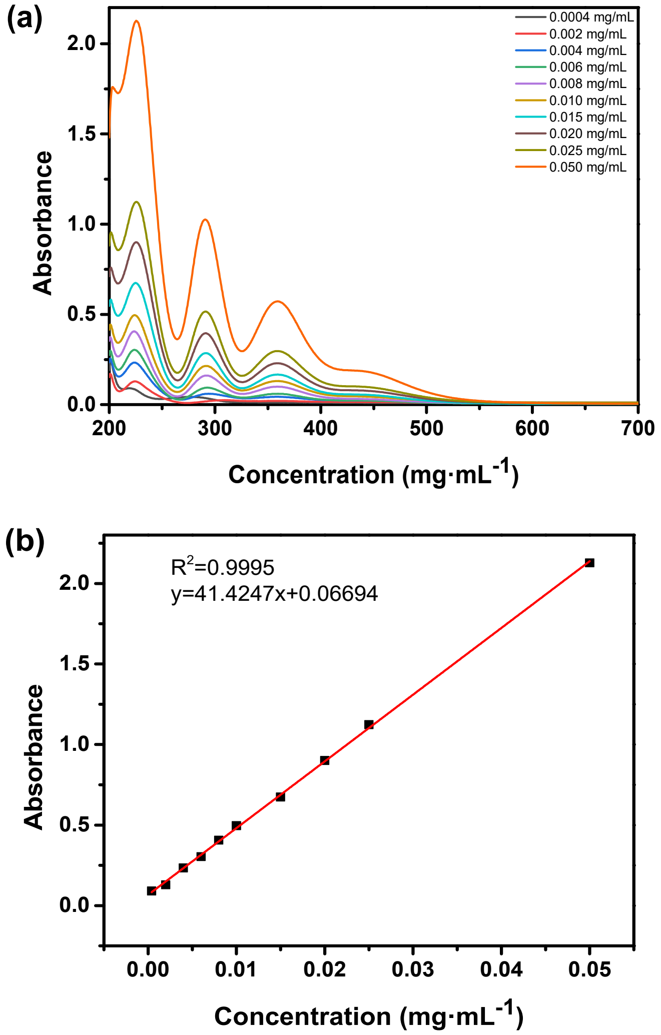

Figure S10. UV-vis spectra of methanol standard solutions of iodine with different concentration (a) and the corresponding calibration curve of absorbance versus iodine concentration established from UV-vis spectra (b) as shown in (a).

Release behavior and reusability measurement.

Typically, the iodine saturated-adsorbed CMP sample (0.4 mg) was treated with methanol (3.0 mL) under stirring for a given time. The mixture was then separated and the collected supernatant was filtered with 0.22 μm Millipore cellulose membrane before UV-vis absorption analysis. The release ratio (x) of the CMPs was calculated as following equation:

$$x = \frac{CV}{m\frac{a}{a+1}} \times 100\% \qquad (2)$$

where, C is the concentration of iodine released into methanol, V is the methanol volume, m is the mass of I_2≅CMP sample, and α represents the iodine adsorption capability.

For reusability measurement, the iodine saturated-adsorbed CMP sample was treated with Soxhlet extraction to remove the iodine from I_2≅TPDA-TFPB-CMP, I_2≅TPDA-TATBA-CMP, and I_2≅TPDA-TECHO-CMP with methanol completely, and the empty samples are dried at 80 °C for 48 hours. The empty CMPs were used for iodine vapor absorption for another adsorption cycle. This same process was repeated for five cycles.

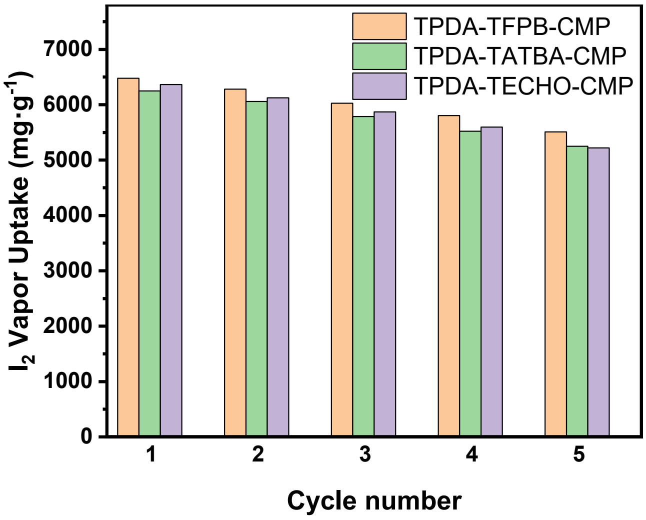

Figure S11. Recyclability of TPDA-TFPB-CMP, TPDA-TATBA-CMP, and TPDA-TECHO-CMP in iodine uptake.

References

1. L. Grunenberg, G. Savasci, M. W. Terban, V. Duppel, I. Moudrakovski, M. Etter, R. E. Dinnebier, C. Ochsenfeld and B. V. Lotsch, J Am Chem Soc, 2021, 143, 3430–3438.
2. N. Wang, L. Feng, X. D. Xu and S. Feng, Macromol Rapid Commun, 2022, 43, e2100885.
3. Y. Xie, T. Pan, Q. Lei, C. Chen, X. Dong, Y. Yuan, J. Shen, Y. Cai, C. Zhou, I. Pinnau and Y. Han, Angew. Chem. Int. Ed. 2021, 60, 22432–22440.
4. J. H. Chang, H. Li, J. Zhao, X. Guan, C. Li, G. Yu, V. Valtchev, Y. Yan, S. Qiu and Q. Fang, Chem Sci, 2021, 12, 8452–8457.
5. X. Guo, Y. Li, M. Zhang, K. Cao, Y. Tian, Y. Qi, S. Li, K. Li, X. Yu and L. Ma, Angew Chem Int Ed, 2020, 59, 22697–22705.
6. P. Wang, Q. Xu, Z. Li, W. Jiang, Q. Jiang and D. Jiang, Adv Mater, 2018, 30, 1801991.
7. J. Li, H. Zhang, L. Zhang, K. Wang, Z. Wang, G. Liu, Y. Zhao and Y. Zeng, J. Mater. Chem. A, 2020, 8, 9523–9527.
8. X. Guo, Y. Tian, M. Zhang, Y. Li, R. Wen, X. Li, X. Li, Y. Xue, L. Ma, C. Xia and S. Li, Chem. Mater, 2018, 30, 2299–2308.
9. T. Geng, S. Ye, Z. Zhu and W. Zhang, J. Mater. Chem. A, 2018, 6, 2808–2816.
10. Z. J. Yin, S. Q. Xu, T. G. Zhan, Q. Y. Qi, Z. Q. Wu and X. Zhao, Chem Commun (Camb), 2017, 53, 7266–7269.
11. S. Luo, Q. Yan, S. Wang, H. Hu, S. Xiao, X. Su, H. Xu and Y. Gao, ACS Appl. Mater. Interfaces, 2023, 15, 46408–46416.
12. Z. Wen, S. Wang, S. Fu, J. Qian, Q. Yan, H. Xu, K. Zuo, X. Su, C. Zeng and Y. Gao, Chem Res Chinese U, 2022, 38, 472–477.
13. Z. Guo, P. Sun, X. Zhang, J. Lin, T. Shi, S. Liu, A. Sun and Z. Li, Chem Asian J, 2018, 13, 2046–2053.
14. P. Tian, Z. Ai, H. Hu, M. Wang, Y. Li, X. Gao, J. Qian, X. Su, S. Xiao, H. Xu, F. Lu and Y. Gao, Molecules, 2022, 27, 5161.
15. X. Qian, B. Wang, Z. Q. Zhu, H. X. Sun, F. Ren, P. Mu, C. Ma, W. D. Liang and A. Li, J Hazard Mater, 2017, 338, 224–232.
16. K. Su, W. Wang, B. Li and D. Yuan, ACS Sustain. Chem. Eng., 2018, 6, 17402–17409.

17. S. An, X. Zhu, Y. He, L. Yang, H. Wang, S. Jin, J. Hu and H. Liu, Ind. Eng. Chem. Res, 2019, 58, 10495–10502.
18. Q. Jiang, H. Huang, Y. Tang, Y. Zhang and C. Zhong, Ind. Eng. Chem. Res, 2018, 57, 15114–15121.
19. H. Sun, P. La, Z. Zhu, W. Liang, B. Yang, A. Li, J. Mater. Sci. 2015, 50, 7326.
20. Y. Liao, J. Weber, B. M. Mills, Z. Ren, C. F. J. Faul, Macromolecules 2016, 49, 6322.
21. Qi, B.; Liu, Y.; Zheng, T.; Gao, Q.; Yan, X.; Jiao, Y.; Yang, Y. J. Solid State Chem. 2018, 258, 49–55.
22. H. Li, X. Ding and B. H. Han, Chem. Eur. J, 2016, 22, 11863–11868.
23. Ren, Y. Y.; Zhang, W.; Zhu, Y.; Wang, D. G.; Yu, G.; Kuang, G. C. J. Appl. Polym. Sci. 2018, 135, 46106.
24. Z. Yan, Y. Yuan, Y. Tian, D. Zhang, G. Zhu, Angew. Chem. Int. Ed. 2015, 54, 12733.
25. B. J. Riley, J. Chun, J. V. Ryan, J. Matyas, X. S. Li, D. W. Matson, S. K. Sundaram, D. M. Strachan, J. D. Vienna, RSC Adv. 2011, 1, 1704.
26. Q. Q. Dang, X. M. Wang, Y. F. Zhan and X. M. Zhang, Polymer Chem, 2016, 7, 643–647.
27. Y. Li, W. Chen, W. Hao, Y. Li and L. Chen, ACS Appl. Nano Mater, 2018, 1, 4756–4761.
28. K. S. Subrahmanyam, D. Sarma, C. D. Malliakas, K. Polychronopoulou, B. J. Riley, D. A. Pierce, J. Chun, M. G. Kanatzidis, Chem. Mater. 2015, 27, 2619.
29. Y. Zhu, Y. J. Ji, D. G. Wang, Y. Zhang, H. Tang, X. R. Jia, M. Song, G. Yu and G. C. Kuang, J. Mater. Chem. A, 2017, 5, 6622–6629.
30. X. Qian, Z. Q. Zhu, H. X. Sun, F. Ren, P. Mu,W. D. Liang, L. H. Chen, and A. Li, ACS Appl. Mater. Interfaces, 2016, 8, 21063–21069.
31. P. Chen, X. He, M. Pang, X. Dong, S. Zhao and W. Zhang, ACS Appl. Mater. Interfaces, 2020, 12, 20429–20439.
32. Y. Chen, H. Sun, R. Yang, T. Wang, C. Pei, Z. Xiang, Z. Zhu, W. Liang, A. Li, W. Deng, J. Mater. Chem. A 2015, 3, 87–91.
33. Sigen, Y. Zhang, Z. Li, H. Xia, M. Xue, X. Liu and Y. Mu, Chem. Commun., 2014, 50, 8495–8498.
34. H. Ma, J. J. Chen, L. X. Tan, J. H. Bu, Y. H. Zhu, B. E. Tan, and C. Zhang, ACS Macro Lett., 2016, 5, 1039–1043.
35. D. F. Sava, K. W. Chapman, M. A. Rodriguez, J. A. Greathouse, P. S. Crozier, H. Zhao, P. J. Chupas and T. M. Nenoff, Chem. Mater. 2013, 25, 2591–2596.
36. D. F. Sava, M. A. Rodriguez, K. W. Chapman, P. J. Chupas, J. A. Greathouse, P. S. Crozier and T. M. Nenoff, J. Am. Chem. Soc., 2011, 133, 12398–12401.
37. G. Mehlana, G. Ramon, S. A. Bourne, Microporous Mesoporous Mater. 2016, 231, 21.
38. Y. Zhu, Y. J. Ji, D. G. Wang, Y. Zhang, H. Tang, X. R. Jia, M. Song, G. Yu and G. C. Kuang, Journal of Materials Chemistry A, 2017, 5, 6622–6629.
39. K. C. Park, J. Cho and C. Y. Lee, RSC Advances, 2016, 6, 75478–75481.
40. Y. Chen, H. Sun, R. Yang, T. Wang, C. Pei, Z. Xiang, Z. Zhu, W. Liang, A. Li and W. Deng, J. Mater. Chem. A, 2015, 3, 87–91.
41. C. Pei, T. Ben, S. Xu and S. Qiu, J. Mater. Chem. A, 2014, 2, 7179–7187.
42. P. Mu, H. Sun, T. Chen, W. Zhang, Z. Zhu, W. Liang and A. Li, Macromol Mater Eng, 2017, 302, 1700156.
43. L. Li, R. Chen, Y. Li, T. Xiong and Y. Li, Cellulose, 2020, 27, 5879–5892.
44. D. F. Sava, T. J. Garino, and T. M. Nenoff, Ind. Eng. Chem. Res. 2012, 51, 614–620.
45. Y. Wang, G. A. Sotzing, and R. A. Weiss, Polymer 2006, 47, 2728.
46. M. H. Zeng, Q. X. Wang, Y. X. Tan, S. Hu, H. X. Zhao, L. S. Long, and M. Kurmoo, J. Am. Chem. Soc. 2010, 132, 2561.
47. Q. K. Liu, J. P. Ma, and Y. B. Dong, Chem. Comm. 2011, 47, 7185.
48. Z. Yin, Q. X. Wang, and M. H. Zeng, J. Am. Chem. Soc. 2012, 134, 4857.
49. T. Hasell, M. Schmidtmann, and A. I. Cooper, J. Am. Chem. Soc. 2011, 133, 14920.
50. Z. M. Wang, Y. J. Zhang, T. Liu, M. Kurmoo, and S. Gao, Adv. Funct. Mater. 2007, 17, 1523.
51. B. J. Riley, S. Chong, J. Schmid, J. Marcial, E. T. Nienhuis, M. K. Ber, S. Lee, N. L. Canfield, S. Kim, M. A. Derewinski, and R. K. Motkuri, ACS Appl. Mater. Interfaces 2022, 14, 18439–18452.
52. K. W. Chapman, P. J. Chupas, and T. M. Nenoff, J. Am. Chem. Soc. 2010, 132, 8897.
53. P. Katsoulidis, J. He, and M. G. Kanatzidis, Chem. Mater. 2012, 24, 1937.
54. Haefner, D. Methods of Gas Phase Capture of Iodine from Fuel Reprocessing Off-Gas: A Literature Survey; Idaho National Laboratory: Idaho Falls, ID, USA, 2007.
55. B. F. Abrahams, M. Moylan, S. D. Orchard, and R. Robson, Angew. Chem. Int. Ed. 2003, 42, 1848.

Disclaimer/Publisher's Note: The statements, opinions and data contained in all publications are solely those of the individual author(s) and contributor(s) and not of MDPI and/or the editor(s). MDPI and/or the editor(s) disclaim responsibility for any injury to people or property resulting from any ideas, methods, instructions or products referred to in the content.
